# The Subtotal Removal of a Suprasellar Dermoid Cyst Expanding Toward the Olfactory Nerves: A Case Report

**DOI:** 10.7759/cureus.70151

**Published:** 2024-09-25

**Authors:** Fernando Muñoz-Hernandez, Alberto Gallardo, Esther Granell

**Affiliations:** 1 Neurosurgery Department, Hospital Santa Creu i Sant Pau, Barcelona, ESP; 2 Pathological Anatomy Department, Hospital Santa Creu i Sant Pau, Barcelona, ESP; 3 Radiology Department, Hospital Santa Creu i Sant Pau, Barcelona, ESP

**Keywords:** clinical decision-making, congenital dermoid cyst, incidental radiological finding, neurosurgical procedures, suprasellar tumor

## Abstract

Dermoid cysts (DCs) are benign congenital lesions that originate in the ectoderm cells produced during the formation of the neural tube. They are usually located at the cerebral midline, and, on rare occasions, at the suprasellar level. In this case report, we present a 17-year-old female patient with minimal symptoms (hyposmia) caused by a suprasellar dermoid cyst extending toward the anterior cranial fossa and the olfactory nerves with subsequent frontotemporal craniotomy and the subtotal removal of the tumor. Given the rare features of this case, the chosen surgical strategy achieved tumor subtotal resection and symptom remission.

## Introduction

Congenital dermoid cysts (DCs) are formed by remnants of ectodermal cells that become entrapped along the midline of the neural tube during the third to fifth weeks of embryonic development [1-3]. These very rare primary cerebral tumors (less than 1% of all cerebral tumors) are usually benign, asymptomatic, and only diagnosed by chance [1-4]. However, in some cases, their continuous slow-growing expansion can cause symptoms due to the compression of the surrounding area, vasculature, and nerves [2] or when they rupture [5-8]. DCs are commonly located in the posterior fossa, but in extraordinarily rare cases, they can be found in the suprasellar area [3,4].

The case presented in this article is especially interesting due to the fact that the suprasellar DC was expanding toward the anterior fossa and the olfactory nerves, causing hyposmia, and threatening to compress the optic chiasm and right optic nerve.

## Case presentation

A 16-year-old female patient (17 years old by the time of surgery) was referred to our department from her local hospital with a primary diagnosis of dermoid cyst. The cyst was casually discovered through MRI after an episode of fainting. The MRI showed a 4 cm-diameter lesion on the suprasellar area, above the optic chiasm, and a smaller subrostral corpus callosum fat component.

Upon neurological examination, no abnormalities were found; however, the patient reported hyposmia after years of evolution. Despite that the cyst was reaching the optic chiasm and contacting the pituitary stalk, the neuro-ophthalmological examination was normal without any affectation of the visual field or visual acuity, and the hormone analysis did not reveal any alteration. Since the cysts seemed benign and produced no symptoms, the risk of surgery was considered unnecessary, and regular MRI controls were scheduled to assess the evolution of the lesion.

Three months later, the patient reported no new symptoms; however, the MRI reported an increased volume of the lesion and internal changes that could be related to an increased risk of rupture. Given the growth of the cyst and the increased risk of rupture, which could cause serious consequences, the therapeutical approach was changed, and the patient was considered a candidate for surgery. During the preparation for surgery, upon revision of the three-month MRI, the most likely diagnosis was a dermoid cyst on the anterior fossa.

**Figure 1:**
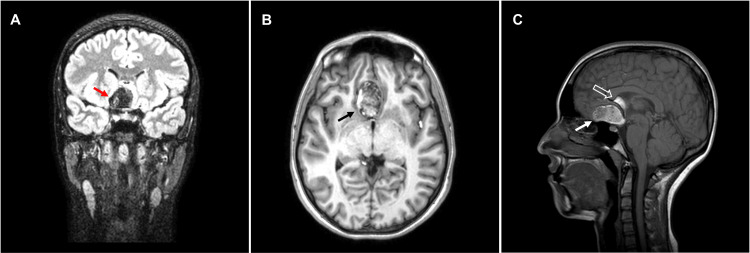
Preoperative MRI (A) Coronal FLAIR at the level of the pituitary stalk showing the suprasellar dermoid cyst (full arrow), (B) axial T1 view of the cyst (full arrow), and (C) sagittal T1 view showing both the suprasellar dermoid cyst (full arrow) and the subrostral corpus callosum fatty component (empty arrow) FLAIR: fluid-attenuated inversion recovery

Six months later, prior to surgery, a new MRI confirmed a dermoid cyst as the primary diagnosis (Figure 1). The lesion was 38×24×23 mm with well-defined margins. The lesion was heterogeneous and mainly hyperintense on T1 and hyperintense on T2 and did not show restricted diffusion. The mass effect of the lesion was pressuring the adjacent parenchyma and the gyrus rectus, displacing the midline by 9 mm. As previously stated, the lesion was pushing the pituitary stalk posteriorly and the optic chiasm inferiorly. Multiple hyperintense signals were observed in the subarachnoid space of the frontal end of the insular sulcus and the left Sylvian fissure that could be related to the cystic material released due to its rupture.

Two surgical strategies were proposed: the transcranial anterolateral approach and the transplanum-transtuberculum endoscopic approach. Finally, a transcranial approach was decided, to increase the chances of preservation and perhaps the improvement of smell. In addition, the endonasal endoscopic route increased the risk of cerebrospinal fluid (CSF) leak compared to the transcranial route.

As shown in Video 1, the patient was placed in a supine position with her head tilted to the left about 15°. The incision was frontotemporal behind the hairline, 1 cm above the zygomatic bone, and at a superior level, it reached the midline. After the subcutaneous dissection and subfascial dissection of the temporal muscle, a frontotemporal craniotomy was performed, exposing more of the frontal lobe than the temporal lobe. The burr hole was made on the posterior part of the craniotomy with the intention of minimizing the possible aesthetic defect that usually occurs at the pterion area. The drilling of the pterion, the lesser wing of the sphenoid bone, up to the superior orbital fissure was performed. The dural opening was initially carried out following the line of the Sylvian fissure to later open in a triangle at the level of the pterion. The frontal and temporal lobes were protected by the dura mater at all times. An initial subfrontal approach was performed to release CSF from the perichiasmatic cisterns. The presence of hairs inside the tumor during its extirpation was in line with the dermoid cyst diagnosis and ruled out the epidermoid cyst alternative diagnosis. The subtotal removal of the tumor was performed, leaving minimal remains of the dermoid cyst adhered to the capsule that was in intimate contact with the pia mater and the vessels of the circle of Willis. A final inspection with a 30° endoscope was performed, confirming the almost complete removal of the lesion. A standard closure of the craniotomy was performed.

**Video 1:** Surgical intervention The transcranial anterolateral subtotal resection of the dermoid cyst

**Figure 2:**
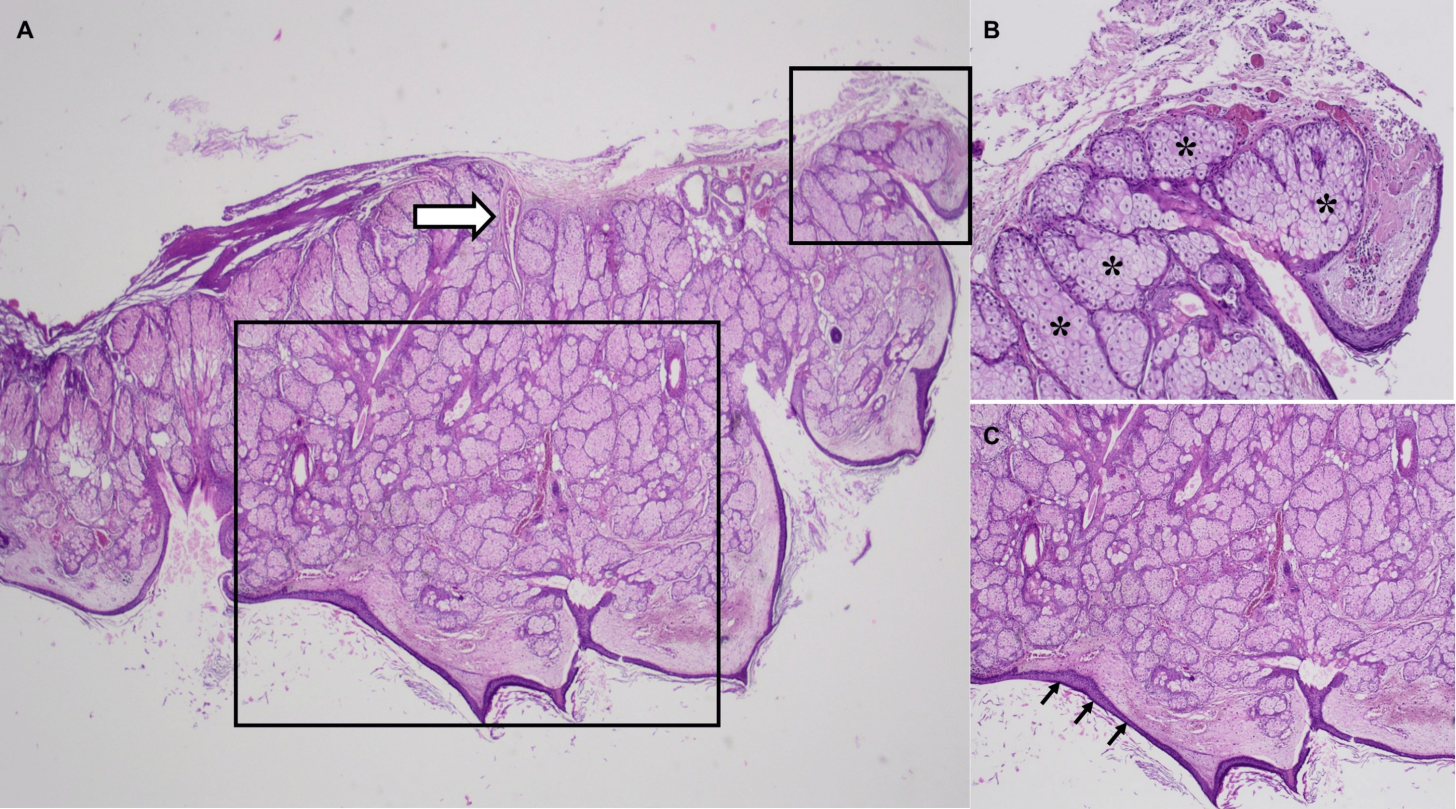
Histological preparations of the extracted material (A) 20× hematoxylin and eosin preparation of the extracted material showing a general view of the cyst including some hair follicles (thick arrow), (B) 80× hematoxylin and eosin preparation of the extracted material showing the detail of abundant sebaceous glands of the cyst (asterisks), and (C) 40× hematoxylin and eosin preparation of the extracted material showing a detail of the epithelium covering the cyst (small arrows)

The extracted material was sent for histopathological analysis, which confirmed the diagnosis of dermoid cyst and the lack of malignity (Figure 2).

**Figure 3:**
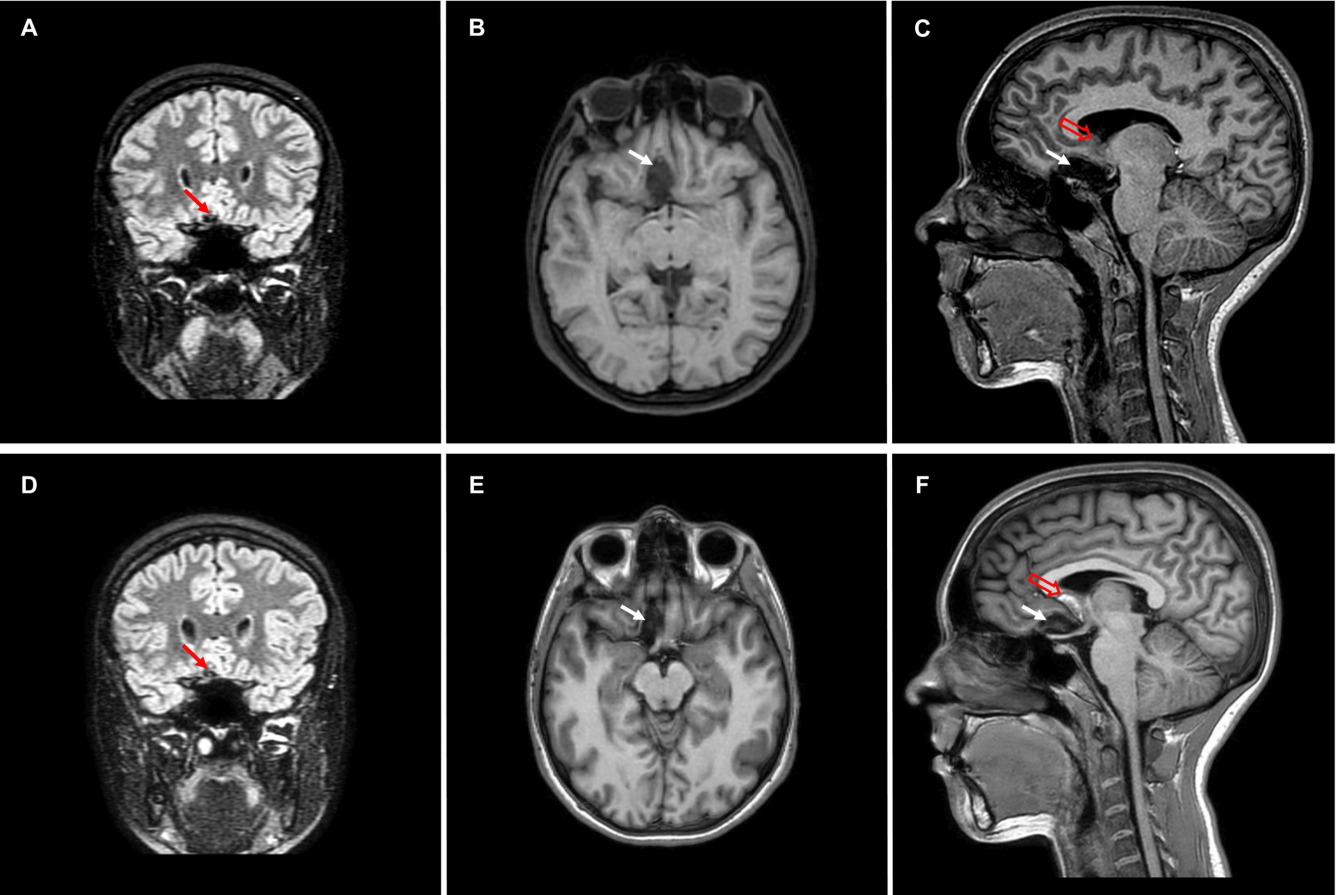
Postoperative MRI progressive changes within the surgical cavity (full arrows) and a slight decrease of the subrostral component under the corpus callosum (empty arrows) (A) One-year postoperative FLAIR coronal view, (B) one-year postoperative T1 axial view, (C) one-year postoperative T1 sagittal view, (D) two-year postoperative FLAIR coronal view, (E) two-year postoperative T1 axial view, and (F) two-year postoperative T1 sagittal view FLAIR: fluid-attenuated inversion recovery

No complications or neurological sequelae were observed during the postoperative period. One month after surgery, during a follow-up visit, the patient reported recovery of her sense of smell. The patient was followed up with an MRI three months, one year, and two years after surgery. Between three months and two years after surgery, the size of the postsurgical cavity continuously decreased in size, and the subrostral component of the corpus callosum showed a slight decrease in size in the follow-up assessments (16.5×6.3×6.5 mm in 2023 versus 18.4×7.8×10 mm in 2021) (Figure 3).

Prior to the preparation of this case report, the patient was contacted and asked permission to publish her case and images obtained during her treatment. She was also informed that no identifiable personal information would be disclosed in this publication.

## Discussion

Dermoid cysts are considered a benign type of brain tumor that are usually asymptomatic [5-7]. However, in some cases, due to their slow-growing expansion, they can cause symptoms such as headaches and seizures by their own mass effect [3]. In the case exposed here, its anterior fossa expansion was pressuring against the olfactory nerve causing hyposmia, and there was a certain risk that further growth toward the optic chiasm and optic nerve could lead to vision impairment. In previously reported cases, vision impairments were caused by the rupture of a DC located close to the optic nerve or optic chiasm, and in all these cases, they had to be removed through surgery [5,6,9]. This is why we preemptively decided to surgically remove the DC to avoid its uncontrolled rupture and more serious complications.

The treatment of suprasellar DCs is not always straightforward. There are many potential approaches that can be taken, from conservative treatment with long-term follow-up [10] to different surgical approaches such as the endonasal endoscopic approach or accessing the tumor through craniotomy [2,4,5,11]. General consensus has usually been to intervene when the cyst ruptures or it is pressuring nearby areas and causing symptoms, although complete removal is not always possible [2,8,11,12]. To decide whether to approach through craniotomy or endonasal endoscopy, it is important to take into account the location, size, and affected areas near the cyst. In this case, upon the results of the follow-up MRI showing growth and increased risk of rupture, to preserve the olfactory nerves and attempt to reverse the hyposmia caused by the cyst and also to prevent CSF leakage, the surgical approach of choice was a frontotemporal craniotomy. The clinical evolution of the patient, with smell sense recovery, confirmed the success of the approach.

The main limitation of this article is that it only covers the experience of one case. However, due to the limited number of suprasellar cysts described in the literature, the author considered this to be an interesting case to showcase the importance of an adequate surgical approach to achieve the maximum benefit while reducing the risks when dealing with such an unusual case.

## Conclusions

The management of suprasellar dermoid cysts requires the careful consideration of various factors. In this specific case, the tumor's mild symptoms were initially managed conservatively, but its growth toward the optic chiasm indicated a need for surgical intervention. Among the available surgical options, a frontotemporal craniotomy was deemed the most suitable approach to preserve the patient's smell and minimize the risk of CFS leakage compared to an endoscopic endonasal approach. The take-home message from this case is that despite their generally benign nature, dermoid cysts require follow-up to identify the risk of rupture or new symptoms caused by its growth, and if needed, the surgical approach must take into account the surrounding structures and potential risks of each option.
